# (*E*)-Methyl *N*′-(4-bromo­benzyl­idene)­hydrazinecarboxyl­ate at 123 K

**DOI:** 10.1107/S1600536808021983

**Published:** 2008-07-19

**Authors:** Rong Sun, Xiang-Wei Cheng

**Affiliations:** a Zhejiang Police College Experience Center, Zhejiang Police College, Hangzhou 310053, People’s Republic of China

## Abstract

The title compound, C_9_H_9_BrN_2_O_2_, crystallizes with two independent but essentially identical mol­ecules in the asymmetric unit. Each mol­ecule adopts a *trans* configuration with respect to the C=N bond. In one of the mol­ecules, the dihedral angle between the benzene ring and the hydrazinecarboxylic acid plane is 24.9 (2)°, and that in the other mol­ecule is 16.1 (2)°. The mol­ecules are linked into a three-dimensional network *via* inter­molecular N—H⋯O, C—H⋯O, C—H⋯N and C—H⋯Br hydrogen bonds. An intramolecular N—H⋯O hydrogen bond is also present.

## Related literature

For general background, see: Parashar *et al.* (1988 ▶); Hadjoudis *et al.* (1987 ▶); Borg *et al.* (1999 ▶). For a related structure, see: Cheng (2008 ▶).
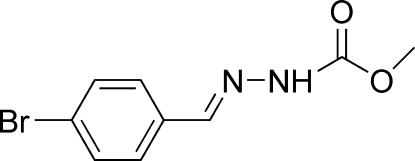


## Experimental

### 

#### Crystal data

C_9_H_9_BrN_2_O_2_
*M* *_r_* = 257.09
Monoclinic, 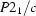

*a* = 13.8585 (10) Å
*b* = 9.5257 (7) Å
*c* = 15.5871 (11) Å
β = 95.967 (3)°
*V* = 2046.5 (3) Å^3^
*Z* = 8
Mo *K*α radiation
μ = 3.99 mm^−1^
*T* = 123 (2) K
0.30 × 0.26 × 0.25 mm

#### Data collection

Bruker SMART CCD area-detector diffractometer
Absorption correction: multi-scan (*SADABS*; Bruker, 2002 ▶) *T*_min_ = 0.320, *T*_max_ = 0.367
20978 measured reflections
3610 independent reflections
2615 reflections with *I* > 2σ(*I*)
*R*_int_ = 0.056

#### Refinement

*R*[*F* ^2^ > 2σ(*F* ^2^)] = 0.036
*wR*(*F* ^2^) = 0.099
*S* = 1.05
3610 reflections
254 parameters
H-atom parameters constrained
Δρ_max_ = 0.76 e Å^−3^
Δρ_min_ = −0.49 e Å^−3^

### 

Data collection: *SMART* (Bruker, 2002 ▶); cell refinement: *SAINT* (Bruker, 2002 ▶); data reduction: *SAINT*; program(s) used to solve structure: *SHELXS97* (Sheldrick, 2008 ▶); program(s) used to refine structure: *SHELXL97* (Sheldrick, 2008 ▶); molecular graphics: *SHELXTL* (Sheldrick, 2008 ▶); software used to prepare material for publication: *SHELXTL*.

## Supplementary Material

Crystal structure: contains datablocks I, global. DOI: 10.1107/S1600536808021983/ci2632sup1.cif

Structure factors: contains datablocks I. DOI: 10.1107/S1600536808021983/ci2632Isup2.hkl

Additional supplementary materials:  crystallographic information; 3D view; checkCIF report

## Figures and Tables

**Table 1:** Hydrogen-bond geometry (Å, °)

*D*—H⋯*A*	*D*—H	H⋯*A*	*D*⋯*A*	*D*—H⋯*A*
N2—H2*A*⋯O3^i^	0.88	2.01	2.854 (4)	160
N4—H4*A*⋯O1	0.88	2.04	2.896 (3)	165
C7—H7⋯O3^i^	0.95	2.49	3.261 (4)	139
C9—H9*A*⋯Br2^ii^	0.98	2.89	3.697 (3)	141
C18—H18*C*⋯N1^iii^	0.98	2.60	3.548 (5)	162

Symmetry codes: (i) 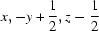; (ii) 
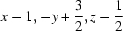
; (iii) 
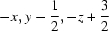
.

**Fig. 1.**
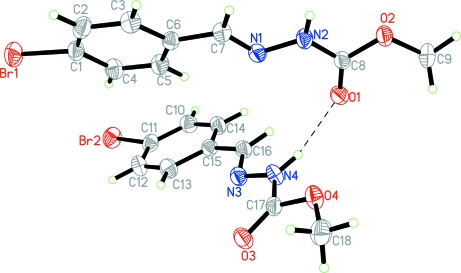
Molecular structure of the title compound, showing 30% probability displacement ellipsoids and the atomic numbering.

**Fig. 2.**
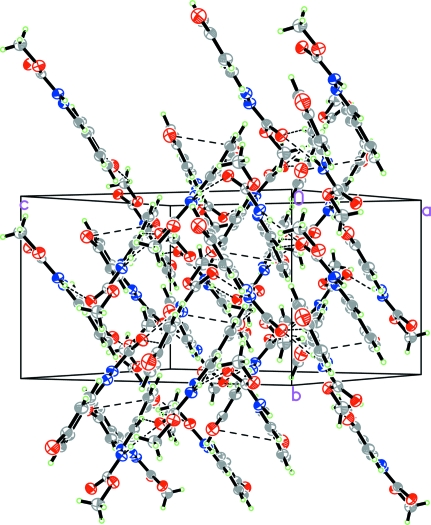
The crystal packing of the title compound. Hydrogen bonds are shown as dashed lines.

## supplementary crystallographic information

### Comment

Benzaldehydehydrazone derivatives have received considerable attention for a long time due to their pharmacological activity (Parashar *et al.*, 1988) and their photochromic properties (Hadjoudis *et al.*, 1987). They are important intermidiates for 1,3,4-oxadiazoles, which have been reported to be versatile compounds with many properties (Borg *et al.*, 1999). As a further investigation of this type of derivatives, the crystal structure of the title compound is reported here.

The title molecule (Fig.1) crystallizes with two independent but essentially identical molecules in the asymmetric unit. Each independent molecule adopts a *trans* configuration with respect to the C═N bond. In each molecule, the hydrazine carboxylic acid methyl ester group is twisted away from the attached ring. The dihedral angle between C1-C6 and N1/N2/O1/O2/C7-C9 planes is 24.9 (2)° and that between C10-C15 and N3/N4/O3/O4/C16-C18 planes is 14.8 (2)°. The bond lengths and angles of each molecule in the asymmetric unit agree with those observed for ethyl N'-[(E)-4-hydroxybenzylidene]hydrazinecarboxylate (Cheng, 2008). The independent molecules are linked through N4-H4A···O1 hydrogen bond.

The molecules are linked into a three-dimensional network by intermolecular N—H···O, C—H···O, C—H···N and C—H···Br hydrogen bonds (Fig.2).

### Experimental

4-Bromobenzaldehyde (1.84 g, 0.01 mol) and methyl hydrazinecarboxylate (0.90 g, 0.01 mol) were dissolved in stirred methanol (25 ml) and left for 3 h at room temperature. The resulting solid was filtered off and recrystallized from ethanol to give the title compound in 75% yield. Single crystals suitable for X-ray analysis were obtained by slow evaporation of a methanol solution at room temperature (m.p. 459–462 K).

### Refinement

H atoms were positioned geometrically (N-H = 0.88 Å and C-H = 0.95 or 0.98 Å) and refined using a riding model, with *U*_iso_(H) = 1.2–1.5*U*_eq_(C).

### Crystal data

**Table:**

C_9_H_9_BrN_2_O_2_	*F*_000_ = 1024
*M**_r_* = 257.09	*D*_x_ = 1.669 Mg m^−^^3^
Monoclinic, *P*2_1_/*c*	Mo *K*α radiation λ = 0.71073 Å
Hall symbol: -P 2ybc	Cell parameters from 3610 reflections
*a* = 13.8585 (10) Å	θ = 1.5–25.0º
*b* = 9.5257 (7) Å	µ = 3.99 mm^−^^1^
*c* = 15.5871 (11) Å	*T* = 123 (2) K
β = 95.967 (3)º	Block, colourless
*V* = 2046.5 (3) Å^3^	0.30 × 0.26 × 0.25 mm
*Z* = 8	

### Data collection

**Table:**

Bruker SMART CCD area-detector diffractometer	3610 independent reflections
Radiation source: fine-focus sealed tube	2615 reflections with *I* > 2σ(*I*)
Monochromator: graphite	*R*_int_ = 0.056
*T* = 123(2) K	θ_max_ = 25.0º
φ and ω scans	θ_min_ = 1.5º
Absorption correction: multi-scan(SADABS; Bruker, 2002)	*h* = −16→16
*T*_min_ = 0.320, *T*_max_ = 0.368	*k* = −11→9
20978 measured reflections	*l* = −18→18

### Refinement

**Table:**

Refinement on *F*^2^	Hydrogen site location: inferred from neighbouring sites
Least-squares matrix: full	H-atom parameters constrained
*R*[*F*^2^ > 2σ(*F*^2^)] = 0.036	*w* = 1/[σ^2^(*F*_o_^2^) + (0.048*P*)^2^ + 0.8965*P*] where *P* = (*F*_o_^2^ + 2*F*_c_^2^)/3
*wR*(*F*^2^) = 0.099	(Δ/σ)_max_ = 0.001
*S* = 1.05	Δρ_max_ = 0.76 e Å^−^^3^
3610 reflections	Δρ_min_ = −0.49 e Å^−^^3^
254 parameters	Extinction correction: SHELXL97 (Sheldrick, 2008), Fc^*^=kFc[1+0.001xFc^2^λ^3^/sin(2θ)]^-1/4^
Primary atom site location: structure-invariant direct methods	Extinction coefficient: 0.0078 (6)
Secondary atom site location: difference Fourier map	

### Special details

**Table:**

Geometry. All e.s.d.'s (except the e.s.d. in the dihedral angle between two l.s. planes) are estimated using the full covariance matrix. The cell e.s.d.'s are taken into account individually in the estimation of e.s.d.'s in distances, angles and torsion angles; correlations between e.s.d.'s in cell parameters are only used when they are defined by crystal symmetry. An approximate (isotropic) treatment of cell e.s.d.'s is used for estimating e.s.d.'s involving l.s. planes.
Refinement. Refinement of *F*^2^ against ALL reflections. The weighted *R*-factor *wR* and goodness of fit *S* are based on *F*^2^, conventional *R*-factors *R* are based on *F*, with *F* set to zero for negative *F*^2^. The threshold expression of *F*^2^ > σ(*F*^2^) is used only for calculating *R*-factors(gt) *etc*. and is not relevant to the choice of reflections for refinement. *R*-factors based on *F*^2^ are statistically about twice as large as those based on *F*, and *R*- factors based on ALL data will be even larger.

### Fractional atomic coordinates and isotropic or equivalent isotropic displacement parameters (Å^2^)

**Table:**

	*x*	*y*	*z*	*U*_iso_*/*U*_eq_	
Br2	0.54645 (3)	0.79291 (5)	0.97542 (3)	0.07171 (19)	
Br1	0.53903 (3)	0.13944 (5)	0.80455 (3)	0.0767 (2)	
O2	−0.13460 (16)	0.5200 (3)	0.56109 (14)	0.0519 (6)	
O1	−0.05639 (17)	0.5733 (3)	0.69189 (14)	0.0545 (6)	
O3	−0.01087 (16)	0.2800 (3)	0.95955 (15)	0.0562 (6)	
O4	−0.12520 (16)	0.3158 (3)	0.84755 (15)	0.0558 (6)	
N2	−0.00584 (19)	0.3978 (3)	0.60735 (17)	0.0473 (7)	
H2A	−0.0224	0.3431	0.5628	0.057*	
N4	0.0057 (2)	0.4480 (3)	0.85778 (17)	0.0484 (7)	
H4A	−0.0223	0.4915	0.8120	0.058*	
N3	0.09646 (19)	0.4870 (3)	0.89337 (16)	0.0473 (7)	
N1	0.07984 (19)	0.3755 (3)	0.65906 (16)	0.0447 (7)	
C8	−0.0638 (2)	0.5048 (4)	0.6263 (2)	0.0423 (7)	
C6	0.2240 (2)	0.2373 (3)	0.6832 (2)	0.0420 (7)	
C7	0.1282 (2)	0.2684 (4)	0.6391 (2)	0.0443 (8)	
H7	0.1012	0.2075	0.5946	0.053*	
C16	0.1337 (2)	0.5923 (4)	0.8575 (2)	0.0473 (8)	
H16	0.0977	0.6392	0.8109	0.057*	
C14	0.2665 (3)	0.7650 (4)	0.8564 (2)	0.0527 (9)	
H14	0.2265	0.8183	0.8154	0.063*	
C3	0.2686 (3)	0.1104 (4)	0.6674 (2)	0.0519 (9)	
H3	0.2350	0.0434	0.6304	0.062*	
C17	−0.0398 (2)	0.3416 (4)	0.8945 (2)	0.0435 (8)	
C15	0.2313 (2)	0.6402 (3)	0.8879 (2)	0.0449 (8)	
C18	−0.1841 (3)	0.2064 (4)	0.8805 (3)	0.0655 (10)	
H18A	−0.2442	0.1955	0.8421	0.098*	
H18B	−0.1998	0.2321	0.9384	0.098*	
H18C	−0.1481	0.1177	0.8835	0.098*	
C4	0.3665 (2)	0.3015 (4)	0.7766 (2)	0.0519 (9)	
H4	0.4004	0.3664	0.8150	0.062*	
C2	0.3607 (3)	0.0802 (4)	0.7048 (2)	0.0566 (9)	
H2	0.3904	−0.0069	0.6937	0.068*	
C5	0.2746 (3)	0.3315 (4)	0.7396 (2)	0.0508 (9)	
H5	0.2450	0.4178	0.7526	0.061*	
C13	0.2918 (3)	0.5639 (4)	0.9480 (2)	0.0552 (9)	
H13	0.2691	0.4785	0.9703	0.066*	
C10	0.3585 (3)	0.8122 (4)	0.8839 (2)	0.0562 (9)	
H10	0.3815	0.8979	0.8625	0.067*	
C11	0.4167 (2)	0.7338 (4)	0.9426 (2)	0.0521 (9)	
C1	0.4090 (2)	0.1767 (4)	0.7578 (2)	0.0484 (8)	
C9	−0.2049 (3)	0.6282 (4)	0.5732 (3)	0.0651 (10)	
H9A	−0.2534	0.6314	0.5228	0.098*	
H9B	−0.1721	0.7192	0.5802	0.098*	
H9C	−0.2371	0.6073	0.6248	0.098*	
C12	0.3837 (3)	0.6103 (4)	0.9756 (2)	0.0575 (9)	
H12	0.4241	0.5578	1.0168	0.069*	

### Atomic displacement parameters (Å^2^)

**Table:**

	*U*^11^	*U*^22^	*U*^33^	*U*^12^	*U*^13^	*U*^23^
Br2	0.0498 (2)	0.0817 (3)	0.0820 (3)	−0.0070 (2)	−0.00095 (19)	−0.0060 (2)
Br1	0.0418 (2)	0.1006 (4)	0.0865 (3)	0.0154 (2)	0.00182 (18)	0.0141 (2)
O2	0.0420 (12)	0.0612 (15)	0.0504 (13)	0.0084 (11)	−0.0059 (10)	−0.0043 (11)
O1	0.0605 (15)	0.0590 (15)	0.0427 (13)	0.0140 (12)	−0.0011 (11)	−0.0030 (12)
O3	0.0524 (14)	0.0644 (17)	0.0493 (14)	0.0002 (12)	−0.0066 (11)	0.0131 (12)
O4	0.0439 (13)	0.0578 (15)	0.0625 (14)	−0.0003 (11)	−0.0102 (11)	0.0068 (12)
N2	0.0409 (15)	0.0501 (17)	0.0487 (15)	0.0057 (13)	−0.0060 (12)	−0.0100 (13)
N4	0.0487 (16)	0.0476 (18)	0.0459 (15)	0.0009 (13)	−0.0096 (12)	0.0090 (13)
N3	0.0452 (16)	0.0499 (18)	0.0453 (15)	0.0034 (13)	−0.0025 (12)	−0.0006 (13)
N1	0.0415 (15)	0.0497 (18)	0.0420 (14)	0.0004 (13)	−0.0005 (11)	0.0008 (12)
C8	0.0357 (16)	0.049 (2)	0.0417 (18)	−0.0013 (15)	0.0014 (13)	0.0047 (16)
C6	0.0416 (17)	0.0395 (19)	0.0449 (17)	0.0017 (15)	0.0045 (13)	0.0022 (14)
C7	0.0448 (18)	0.040 (2)	0.0470 (18)	−0.0026 (15)	0.0012 (14)	−0.0019 (15)
C16	0.052 (2)	0.045 (2)	0.0430 (18)	0.0084 (16)	0.0009 (15)	−0.0022 (16)
C14	0.057 (2)	0.048 (2)	0.052 (2)	0.0085 (17)	−0.0007 (16)	0.0052 (17)
C3	0.055 (2)	0.043 (2)	0.057 (2)	−0.0030 (17)	0.0008 (16)	−0.0047 (16)
C17	0.0405 (17)	0.045 (2)	0.0437 (18)	0.0097 (15)	−0.0005 (14)	−0.0026 (15)
C15	0.0474 (18)	0.043 (2)	0.0436 (17)	0.0068 (16)	0.0022 (14)	−0.0030 (15)
C18	0.048 (2)	0.070 (3)	0.078 (3)	−0.005 (2)	−0.0002 (18)	0.001 (2)
C4	0.0491 (19)	0.048 (2)	0.056 (2)	−0.0009 (17)	−0.0036 (16)	−0.0030 (16)
C2	0.055 (2)	0.049 (2)	0.068 (2)	0.0130 (18)	0.0124 (18)	0.0032 (18)
C5	0.053 (2)	0.043 (2)	0.055 (2)	0.0035 (16)	−0.0003 (16)	−0.0064 (16)
C13	0.061 (2)	0.045 (2)	0.058 (2)	0.0010 (18)	0.0006 (17)	0.0061 (17)
C10	0.059 (2)	0.052 (2)	0.058 (2)	−0.0010 (18)	0.0059 (17)	0.0058 (17)
C11	0.0416 (18)	0.057 (2)	0.057 (2)	0.0005 (17)	0.0024 (15)	−0.0102 (17)
C1	0.0379 (17)	0.054 (2)	0.0537 (19)	0.0032 (16)	0.0041 (15)	0.0096 (17)
C9	0.0423 (19)	0.072 (3)	0.078 (3)	0.0148 (19)	−0.0051 (18)	0.004 (2)
C12	0.054 (2)	0.053 (2)	0.062 (2)	0.0061 (18)	−0.0060 (17)	0.0071 (18)

### Geometric parameters (Å, °)

**Table:**

Br2—C11	1.903 (3)	C14—C15	1.394 (5)
Br1—C1	1.906 (3)	C14—H14	0.95
O2—C8	1.345 (4)	C3—C2	1.377 (5)
O2—C9	1.445 (4)	C3—H3	0.95
O1—C8	1.208 (4)	C15—C13	1.394 (5)
O3—C17	1.204 (4)	C18—H18A	0.98
O4—C17	1.348 (4)	C18—H18B	0.98
O4—C18	1.450 (5)	C18—H18C	0.98
N2—C8	1.349 (4)	C4—C1	1.371 (5)
N2—N1	1.380 (3)	C4—C5	1.374 (5)
N2—H2A	0.88	C4—H4	0.95
N4—C17	1.352 (4)	C2—C1	1.364 (5)
N4—N3	1.372 (4)	C2—H2	0.95
N4—H4A	0.88	C5—H5	0.95
N3—C16	1.283 (4)	C13—C12	1.375 (5)
N1—C7	1.277 (4)	C13—H13	0.95
C6—C5	1.393 (4)	C10—C11	1.376 (5)
C6—C3	1.392 (5)	C10—H10	0.95
C6—C7	1.460 (4)	C11—C12	1.380 (5)
C7—H7	0.95	C9—H9A	0.98
C16—C15	1.458 (5)	C9—H9B	0.98
C16—H16	0.95	C9—H9C	0.98
C14—C10	1.377 (5)	C12—H12	0.95
			
C8—O2—C9	115.3 (3)	O4—C18—H18B	109.5
C17—O4—C18	115.7 (3)	H18A—C18—H18B	109.5
C8—N2—N1	118.9 (3)	O4—C18—H18C	109.5
C8—N2—H2A	120.5	H18A—C18—H18C	109.5
N1—N2—H2A	120.5	H18B—C18—H18C	109.5
C17—N4—N3	118.7 (3)	C1—C4—C5	119.4 (3)
C17—N4—H4A	120.7	C1—C4—H4	120.3
N3—N4—H4A	120.7	C5—C4—H4	120.3
C16—N3—N4	115.4 (3)	C1—C2—C3	119.4 (3)
C7—N1—N2	115.0 (3)	C1—C2—H2	120.3
O1—C8—O2	124.9 (3)	C3—C2—H2	120.3
O1—C8—N2	126.4 (3)	C4—C5—C6	121.0 (3)
O2—C8—N2	108.6 (3)	C4—C5—H5	119.5
C5—C6—C3	117.9 (3)	C6—C5—H5	119.5
C5—C6—C7	122.7 (3)	C12—C13—C15	121.0 (3)
C3—C6—C7	119.5 (3)	C12—C13—H13	119.5
N1—C7—C6	121.4 (3)	C15—C13—H13	119.5
N1—C7—H7	119.3	C14—C10—C11	119.3 (3)
C6—C7—H7	119.3	C14—C10—H10	120.4
N3—C16—C15	120.3 (3)	C11—C10—H10	120.4
N3—C16—H16	119.9	C10—C11—C12	121.1 (3)
C15—C16—H16	119.9	C10—C11—Br2	119.3 (3)
C10—C14—C15	121.1 (3)	C12—C11—Br2	119.5 (3)
C10—C14—H14	119.5	C2—C1—C4	121.3 (3)
C15—C14—H14	119.5	C2—C1—Br1	119.4 (3)
C2—C3—C6	121.1 (3)	C4—C1—Br1	119.3 (3)
C2—C3—H3	119.5	O2—C9—H9A	109.5
C6—C3—H3	119.5	O2—C9—H9B	109.5
O3—C17—O4	124.4 (3)	H9A—C9—H9B	109.5
O3—C17—N4	126.4 (3)	O2—C9—H9C	109.5
O4—C17—N4	109.2 (3)	H9A—C9—H9C	109.5
C13—C15—C14	118.2 (3)	H9B—C9—H9C	109.5
C13—C15—C16	121.8 (3)	C13—C12—C11	119.3 (3)
C14—C15—C16	120.0 (3)	C13—C12—H12	120.3
O4—C18—H18A	109.5	C11—C12—H12	120.3
			
C17—N4—N3—C16	−177.3 (3)	N3—C16—C15—C13	8.7 (5)
C8—N2—N1—C7	176.9 (3)	N3—C16—C15—C14	−171.7 (3)
C9—O2—C8—O1	0.5 (5)	C6—C3—C2—C1	−0.2 (5)
C9—O2—C8—N2	178.0 (3)	C1—C4—C5—C6	0.0 (5)
N1—N2—C8—O1	−11.7 (5)	C3—C6—C5—C4	1.7 (5)
N1—N2—C8—O2	170.8 (3)	C7—C6—C5—C4	−176.8 (3)
N2—N1—C7—C6	174.9 (3)	C14—C15—C13—C12	0.0 (5)
C5—C6—C7—N1	−9.9 (5)	C16—C15—C13—C12	179.6 (3)
C3—C6—C7—N1	171.6 (3)	C15—C14—C10—C11	0.7 (5)
N4—N3—C16—C15	−177.9 (3)	C14—C10—C11—C12	−1.2 (6)
C5—C6—C3—C2	−1.6 (5)	C14—C10—C11—Br2	176.3 (3)
C7—C6—C3—C2	177.0 (3)	C3—C2—C1—C4	2.0 (5)
C18—O4—C17—O3	0.5 (5)	C3—C2—C1—Br1	−176.7 (3)
C18—O4—C17—N4	−178.2 (3)	C5—C4—C1—C2	−1.8 (5)
N3—N4—C17—O3	4.3 (5)	C5—C4—C1—Br1	176.8 (3)
N3—N4—C17—O4	−177.0 (3)	C15—C13—C12—C11	−0.5 (6)
C10—C14—C15—C13	−0.1 (5)	C10—C11—C12—C13	1.1 (6)
C10—C14—C15—C16	−179.7 (3)	Br2—C11—C12—C13	−176.4 (3)

### Hydrogen-bond geometry (Å, °)

**Table:**

*D*—H···*A*	*D*—H	H···*A*	*D*···*A*	*D*—H···*A*
N2—H2A···O3^i^	0.88	2.01	2.854 (4)	160
N4—H4A···O1	0.88	2.04	2.896 (3)	165
C7—H7···O3^i^	0.95	2.49	3.261 (4)	139
C9—H9A···Br2^ii^	0.98	2.89	3.697 (3)	141
C18—H18C···N1^iii^	0.98	2.60	3.548 (5)	162

Symmetry codes: (i) *x*, −*y*+1/2, *z*−1/2; (ii) *x*−1, −*y*+3/2, *z*−1/2; (iii) −*x*, *y*−1/2, −*z*+3/2.
